# Diabetes is accompanied by secretion of pro-atherosclerotic exosomes from vascular smooth muscle cells

**DOI:** 10.1186/s12933-023-01833-4

**Published:** 2023-05-13

**Authors:** Heng Yu, Hunter F. Douglas, Donald Wathieu, Ryan A. Braun, Christine Edomwande, Daniel J. Lightell Jr., Thaidan Pham, Natasha C. Klingenberg, Shelia Pugh Bishop, Damir B. Khismatullin, T. Cooper Woods

**Affiliations:** 1 Department of Biomedical Engineering, Tulane University, New Orleans, LA USA; 2 Department of Physiology, Tulane University School of Medicine, 1430 Tulane Avenue, New Orleans, LA 70112 USA; 3 Department of Medicine, Tulane University School of Medicine, New Orleans, LA, USA

**Keywords:** Exosomes, microRNA, Atherogenesis/atherosclerosis, Endothelial dysfunction, Macrophage activation, Vascular inflammation

## Abstract

**Background:**

Atherosclerosis is a common co-morbidity of type 2 diabetes mellitus. Monocyte recruitment by an activated endothelium and the pro-inflammatory activity of the resulting macrophages are critical components of atherosclerosis. Exosomal transfer of microRNAs has emerged as a paracrine signaling mechanism regulating atherosclerotic plaque development. MicroRNAs-221 and -222 (miR-221/222) are elevated in vascular smooth muscle cells (VSMCs) of diabetic patients. We hypothesized that the transfer of miR-221/222 via VSMC-derived exosomes from diabetic sources (DVEs) promotes increased vascular inflammation and atherosclerotic plaque development.

**Methods:**

Exosomes were obtained from VSMCs, following exposure to non-targeting or miR-221/-222 siRNA (-KD), isolated from diabetic (DVEs) and non-diabetic (NVEs) sources and their miR-221/-222 content was measured using droplet digital PCR (ddPCR). Expression of adhesion molecules and the adhesion of monocytes was measured following exposure to DVEs and NVEs. Macrophage phenotype following exposure to DVEs was determined by measuring mRNA markers and secreted cytokines. Age-matched apolipoprotein-E-deficient mice null (ApoE^−/−^) mice were maintained on Western diet for 6 weeks and received injections of saline, NVEs, NVE-KDs, DVEs or DVE-KDs every other day. Atherosclerotic plaque formation was measured using Oil Red Oil staining.

**Results:**

Exposure of human umbilical vein and coronary artery endothelial cells to DVEs, but not NVEs, NVE-KDs, or DVE-KDs promoted increased intercellular adhesion molecule-1 expression and monocyte adhesion. DVEs but not NVEs, NVE-KDs, or DVE-KDs also promoted pro-inflammatory polarization of human monocytes in a miR-221/222 dependent manner. Finally, intravenous administration of DVEs, but not NVEs, resulted in a significant increase in atherosclerotic plaque development.

**Conclusion:**

These data identify a novel paracrine signaling pathway that promotes the cardiovascular complications of diabetes mellitus.

**Supplementary Information:**

The online version contains supplementary material available at 10.1186/s12933-023-01833-4.

## Background

Diabetes is associated with an accelerated atherosclerosis resulting in a two- to four-fold increased risk of myocardial infarctions and strokes [1, 2]. Early in atherosclerosis, endothelial cells become activated and increase the expression of adhesion molecules on the cell surface. Endothelial activation has been linked to multiple aspects of diabetes mellitus, including hyperglycemia and insulin resistance [3]. The increase in adhesion molecules on the endothelium leads to the recruitment of inflammatory cells, including monocytes to the developing plaque. Recruited monocytes become macrophages, which can adopt both pro-(M1) and anti-(M2) inflammatory phenotypes depending on the local environment [4]. Identifying the mechanism through which diabetes promotes endothelial activation and increased vascular inflammation is needed to prevent the cardiovascular complications of diabetes.

Paracrine signaling via extracellular vesicles between vascular smooth muscle cells (VSMCs), macrophages and endothelial cells (ECs) is active in atherosclerosis [5–7]. Exosomes, a type of extracellular vesicle microparticle, are capable of transmitting intercellular nucleic acids, proteins, and lipids [8]. Among nucleic acids, cell free microRNAs, small 21 nucleotide RNA molecules found in blood and other bodily fluids, have received attention as potential mediators of cardiovascular and metabolic diseases [9–12]. The content of VSMC-derived exosomes is altered with phenotype and regulates vascular remodeling and repair and thus represents a potential mechanism underlying the cardiovascular complications of diabetes [6, 13]. MicroRNA-221-3p and -222-3p (miR-221/222) promote cell proliferation and migration in VSMCs, driving increased intimal thickening through targeting of the cyclin-dependent kinase inhibitor, p27^Kip1^. In contrast, in ECs, where c-Kit is the more abundant target, cell proliferation and migration are inhibited [14]. Thus, increased miR-221/222 in both VSMCs and ECs will promote atherosclerotic plaque formation by increased intimal thickening coupled with an inability to restore a healthy endothelium. We have previously shown these miRs are elevated in the VSMCs of diabetic mice and the arteries of diabetic patients [15–17].

We hypothesized that VSMC-derived exosomes isolated of diabetic origin (DVEs) would exhibit increased miR-221/222 content and that transfer of these miRNAs would promote vascular inflammation and atherosclerotic plaque formation. We report that DVEs promote both EC activation and M1 polarization of macrophages in a miR-221/222 dependent manner. Furthermore, administration of these exosomes promotes increased atherosclerotic plaque formation in the ApoE^−/−^ mouse model of atherosclerosis. Thus, DVEs represent a novel paracrine signaling pathway underlying the cardiovascular complications of diabetes.

## Methods

### Cell culture

Primary human umbilical vein endothelial cells (HUVECs) were obtained from Cell Applications (San Diego, CA). and cultured in medium 200 (Thermo Fisher Scientific, Waltham, MA) supplemented with Low Serum Growth Supplement (LSGS, Thermo Fisher Scientific) and gentamicin/amphotericin B. Human acute monocytic leukemia cells (THP-1) were obtained from ATCC (Manassas, VA). THP-1 cells were maintained in RPMI 1640 (ATCC) medium supplemented with 10% fetal bovine serum (Thermo Fisher Scientific), 1% penicillin/streptomycin (Thermo Fisher Scientific) and 0.05 mM 2-mercaptoethanol (Sigma Aldrich). Human CD14 + monocytes and primary human coronary artery endothelial cells (HCAECs) were obtained from Lonza (Walkersville, MA). HCAECs were cultured in microvascular endothelial cell growth media (EGM-2 MV, Lonza) according to provider’s instructions. Interferon-γ (IFN-γ) and Tumor Necrosis Factor-α (TNF-α) were purchased from R&D Systems (Minneapolis, MN). For knockdown of *QKI*, monocytes were cultured as above and treated with either a non-targeting control oligonucleotide or one three different *QKI* targeting FlexiTube siRNAs (Qiagen, Germantown, MD) for 18 h prior to preparation of total RNA.

### Exosome isolation and treatment

Human coronary artery vascular smooth muscle cells (HCASMCs) isolated from diabetic and non-diabetic donors as well as smooth muscle growth media (SmGM-2) were purchased from Lonza. HCASMCs were subcultured in SmGM-2 in a humidified 5% CO_2_ atmosphere with the media being replaced every ~ 48–72 h. At 50–70% confluency, the media was replaced with SmGM-2 with exosome depleted fetal bovine serum and conditioned media was collected after 48–72 h. Exosomes were isolated from the conditioned media using Total Exosome Isolation Reagent (Thermo Fisher Scientific) according to the manufacturer’s instructions. Exosomes were prepared from HCASMCs isolates from both male and female diabetic and non-diabetic donors with a minimum of 3 isolates from each sex and disease status. Exosome diameter was determined from cryo-electron microscopy images obtained by the Tulane Microscopy Lab with a Hitachi 4800 High-resolution scanning electron microscope. For mechanistic studies, anti-miR miRNA Inhibitor Negative Control #1 was purchased from Thermo Fisher Scientificand fluorescently labelled miR-221 and miR-222 as well as an antisense RNA to miR-222 with a 2′-O-methylation modified backbone (2’OMe-miR222) were synthesized by IDT (Coralville, IA). HCASMCs were transfected using 0.5% HiPerFect Transfection Reagent (Qiagen) at a concentration of 20 nM overnight, as previously described [17]. HCASMCs were then incubated in SmGM-2 supplemented with 5% exosome free FBS for 48 h and the conditioned media was collected for exosome isolation, as above. ECs and macrophages were incubated in the presence of four exosome preparations (Table 1), including exosomes derived from VSMCs of diabetic origin (DVE), exosomes derived from VSMCs of non-diabetic origin (NVE), DVEs isolated from VSMCs after miRNA221/222 knockdown (DVE + KD), and NVEs isolated from VSMCs after miRNA221/222 knockdown (NVE + KD).

**Table 1:** Summary of treatment groups

Group	Exosome source	Diabetic status of VSMC source	Pre-treatment of VSMC
Cell studies			
Ctrl	PBS		
NVE	HCASMCs	Non-Diabetic	Non-targeting oligo
NVE+KD	HCASMCs	Non-Diabetic	miR-221/222 siRNA
DVE	HCASMCs	Diabetic	Non-targeting oligo
DVE+KD	HCASMCs	Diabetic	miR-221/222 siRNA
Animal study			
Ctrl	Saline		
NVE	NONcNZO10 murine VSMCs	Non-Diabetic	None
DVE	NONcNZO10 murine VSMCs	Diabetic	None

VSMC: Vascular smooth muscle cell; Ctrl: Control; PBS: Phosphate buffered saline; HCASMCs: Human coronary artery smooth muscle cells; NVE: Non-diabetic VSMC derived exosome; DVE: Diabetic VSMC derived exosome; +KD: miR-221/222 knockdown

### Static monocyte adhesion assay

HUVECs and HCAECs were seeded into a 96-well plate and incubated to form an endothelial monolayer. The cells were then incubated in the presence of the four exosome preparations, media alone, or TNF-α. After 6 or 24 h, all supernatants were removed from wells and replaced with RPMI 1640 medium containing THP-1 cells with a concentration of 0.5 × 10^6^ cells/ml. After a 10-min incubation, the THP-1 suspension was carefully aspirated, and the wells were washed twice by phosphate buffered saline (PBS) to eliminate non-firmly attached THP-1 cells. The endothelial monolayer was visualized using a 10× objective in an Eclipse TiS inverted microscope (Nikon, Melville, NY ), and images were taken by a  Qimaging Retiga EXi digital CCD camera (Teledyne, Tucson, AZ) at three randomly selected fields. The number of firmly adherent cells were counted.

### Shear flow-induced monocyte adhesion assay

ECs were seeded and grown to form a monolayer in microfluidic flow channels of a BioFlux™ 48-well plate (Fluxion Biosciences, Oakland, CA) coated with 0.1% gelatin. ECs were then treated with exosomes and controls for 6 h or 24 h. THP-1 cells were applied to the endothelium at a concentration of 0.5 × 10^6^ cells/mL in the BioFlux™ 200 microfluidic system (Fluxion Biosciences) under 1.5 dyne/cm^2^ shear stress for 10 min. The channels were washed with PBS to remove unattached cells and attached THP-1 cells were counted in three randomly selected fields in each channel.

### Fluorescence activated cell sorting (FACS) analysis

ECs were grown to confluence and exposed to exosomes and TNF-a, as described above. ECs were detached with Enzyme-Free PBS-based Cell Dissociation Buffer (Thermo Fisher Scientific) and labelled with fluorescein isothiocyanate (FITC)-conjugated mouse IgG1, IgG2b, mouse anti-human CD54 (Intercellular Adhesion Molecule 1, ICAM-1), and CD106 (Vascular Cell Adhesion Molecules 1, VCAM-1) (Thermo Fisher Scientific). Mean fluorescence intensity and percentage of positively stained cells was calculated using the Attune Acoustic Focusing Cytometer (Thermo Fisher Scientific).

### Protein assays

Interleukin-6 (IL-6) concentrations in conditioned media were measured using the Human High-sensitivity IL-6 ELISA assay (Thermo Fisher Scientific) according to manufacturer’s instructions. QKI protein concentrations in total cell lysates were measured using the Human QKI, KH Domain Containing RNA Binding (QKI) ELISA Kit (Abbexa) according to manufacturer’s instructions. For measurement of RhoA activity, HCAECs were seeded in 60 mm tissue culture dishes and treated 24 h with the exosome preparations. RhoA activity was measured using the G-LISA™ RhoA Activation Assay Biochem Kit (Cytoskeleton, Denver, CO), as previously described [18].

### mRNA and macrophage phenotype measurements

Copies of mRNAs associated with the M1 (*VEGF, CCR7, IL1β, CD80*) and M2 (*MRC1, TIMP3, PDGF*) macrophage phenotype [19, 20] as well as *QKI* were measured using droplet digital PCR (ddPCR). A one-step PCR reaction was prepared using Quantitect primers (Qiagen) coupled with Superscript reverse transcriptase (Qiagen) and the QX200 ddPCR EvaGreen Supermix (Bio-Rad, Hercules, CA). Copy numbers per ng were obtained using the QX200 ddPCR system. For macrophage phenotype scoring, we calculated an M1:M2 score previously used to characterize macrophages phenotype in wound debridement samples [20, 21].

### Animal model of atherosclerosis

15 male ApoE^−/−^ mice (n = 5/group) of 6–8 weeks of age were placed on a Western Diet (42% Fat, Harlan Teklad # TD.88137) for 6 weeks. Murine DVEs and NVEs were obtained from abdominal aortic VSMCs obtained from diabetic and non-diabetic NONcNZO10 mice purchased from Jackson Laboratories, as previously described [17]. DVEs and NVEs at 5 × 10^7^ exosomes/dose or saline vehicle were administered every other day via intravenous injection of the tail vein. At 6 weeks, the mice were euthanized, and the aortae were harvested. Plaque area was identified with Oil Red O staining of the aorta. Plaque area was calculated as the total area of Oil Red O staining divided by the total area of the vessel using Adobe Photoshop. All animal studies were performed with approval of the Tulane University’s Institutional Animal Care and Use Committee.

### Statistical analysis

NCSS 12 and SPSS Statistics 27 software was used to perform the statistical analysis. Student’s *t*-test and one-way ANOVA with Tukey’s HSD test were used for binary and multiple comparisons, respectively. In the case of the flow and FACS analyses comparisons were performed with one-way ANCOVA to control for variability between experiments. A p-value < 0.05 was considered significant.

## Results

### VSMCs release exosomes capable of transferring nucleic acids to adjacent cells

**Fig. 1:**
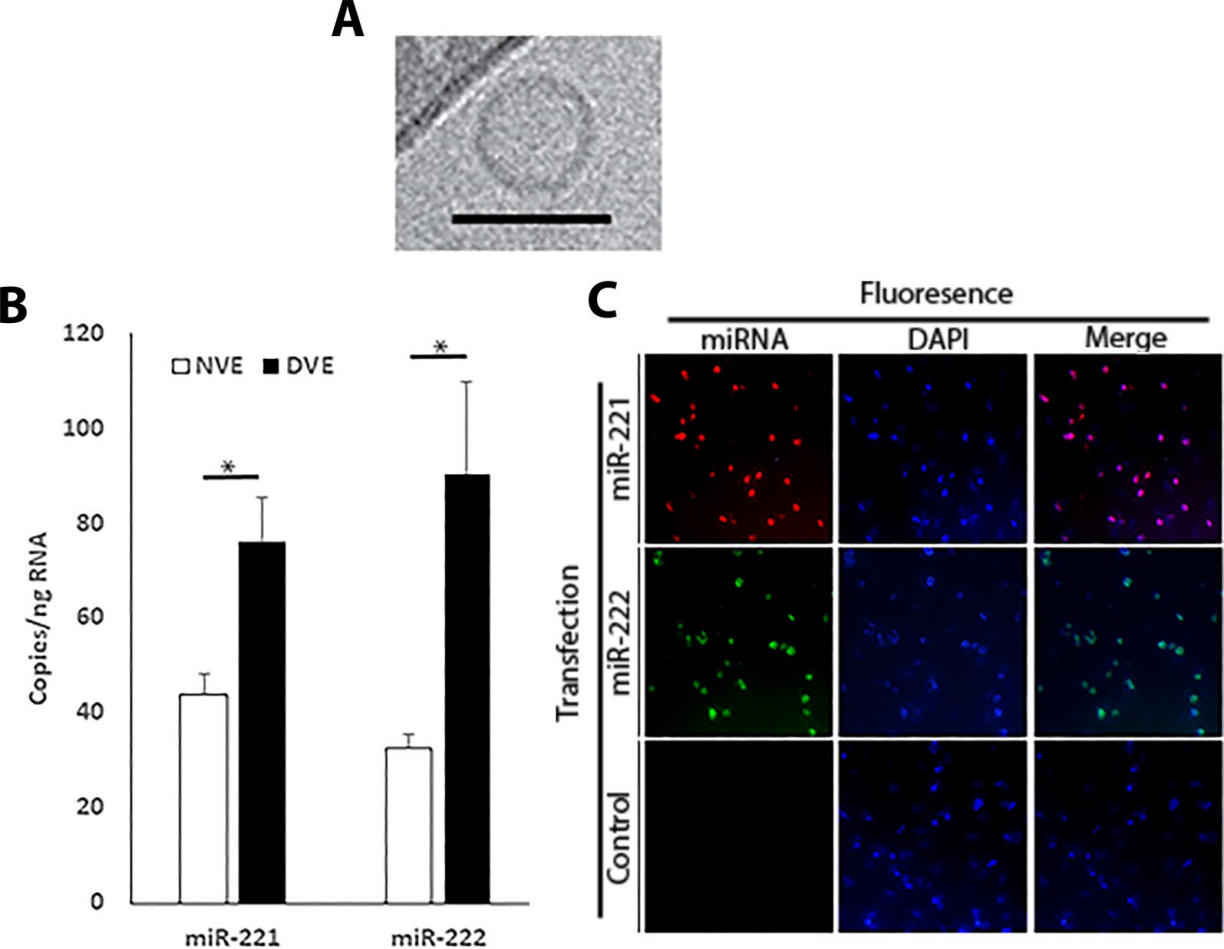
DVEs are exosomes capable of transferring miRNA to from VSMCs to ECs and contain elevated miR-221/222. **A** Cryo-electron microscopy was performed to characterize the extracellular vesicles. The bar represents 50 nm. **B** miR-221/222 content of NVEs and DVEs was measured using real-time qRT-PCR. **C** VSMCs are able to transfer oligonucleotides to adjacent cells. HuCASMCs were transfected with a fluorescent miR-221 (Texas Red) and miR-222 (FITC) for 4 h. HuCASMCs were then trypsinized, washed repeatedly, and seeded into a transwell chamber seated above huCAECs. After 24 h, the huCAECs were fixed and stained with 4′,6-diamidino-2-phenylindole (DAPI). *p < 0.05

We isolated exosomes from VSMCs from donors with and without type 2 diabetes mellitus. Using cryo-electron microscopy (Fig. 1A), we confirmed that the microparticles range in diameter from 30 to 50 nm, agreeing with an exosomes (30–100 nM) [22]. Additionally, we confirmed the vesicles contained two exosomes markers (CD63 and Flotillin, data not shown). Previously, we have reported that miR-221/222 are increased in the arteries of diabetic subjects [16], Fig. 1B shows that this increase is also observed in DVEs compared to NVEs. Finally, Fig. 1C demonstrates the transfer of a fluorescently labelled miR-221 and -222 between human coronary artery ECs and human coronary artery SMCs (huCASMCs) in a transwell chamber. These data confirm that the extracellular vesicles are exosomes capable of transferring miRNA to adjacent cells and that DVEs contain elevated levels of miR-221/222.

### miRNA 221/222 in DVE promote monocyte adhesion on endothelial cells

**Fig. 2:**
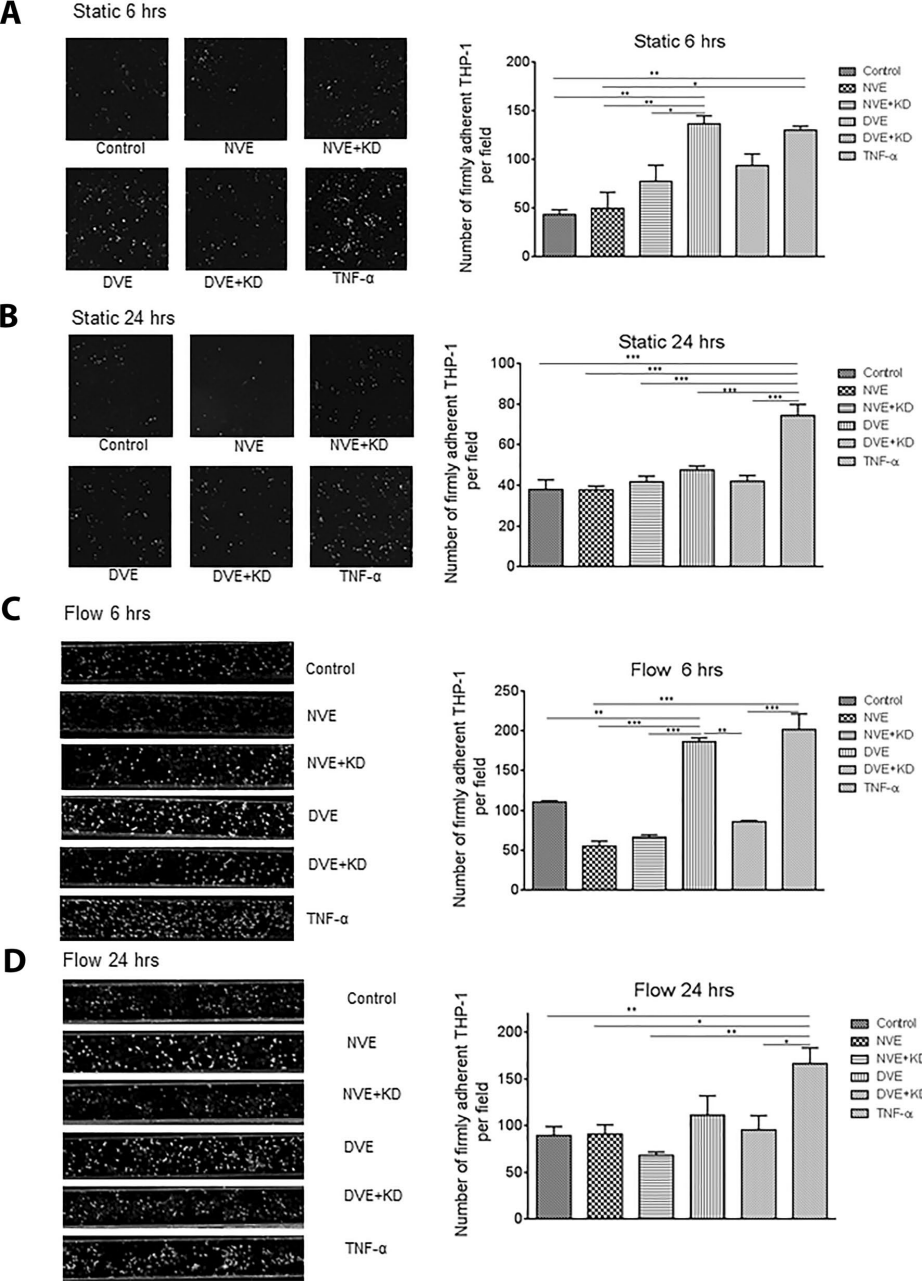
Exposure of HUVECs to DVEs promotes increased adhesion of THP-1 cells under both static and flow conditions. **A**, **B** Number of firmly adherent THP-1 cells on HUVEC under static conditions following 6 (**A**) and 24 (**B**) hours of treatment. **C**, **D** Number of firmly adherent THP-1 cells on HUVEC under shear flow conditions following 6 (**C**) and 24 (**D**) hours of treatment. *p < 0.05, **p < 0.01, ***p < 0.001

To assess the effect of DVEs on the recruitment monocytes by ECs, we measured attachment of THP-1 monocytes to HUVECs and HCAECs under static and flow conditions. The number of adherent THP-1 cells on HUVECs following 6 h treatment with DVEs, but not DVEs + KD, was significantly elevated compared to treatment with vehicle (137 ± 8 vs 34 ± 5, p < 0.01), NVEs (50 ± 16), and NVEs + KD (77 ± 17) and was similar to activation with TNF-α (130 ± 4) (Fig. 2A). This effect was not seen, however, when treatment was prolonged to 24 h (Fig. 2B). Similar effects were seen with HUVECs under shear flow conditions (Fig. 2C). In particular, firm adhesion of THP-1 cells to HUVECs following 6 h exposure to exosomes was significantly increased with the DVE (186 ± 4), and TNF-α (201 ± 20) groups but negligible in the NVE (55 ± 6), NVE + KD (67 ± 3) and DVE + KD (86 ± 2) groups compared to control (110 ± 2). As with static conditions, the effect of exosomes on HUVECs was absent when exposed for 24 h under flow conditions. The data demonstrate that VSMC-derived exosomes of diabetic origin promote increases in HUVEC recruitment of monocytes in a miR221/222 dependent manner.

**Fig. 3:**
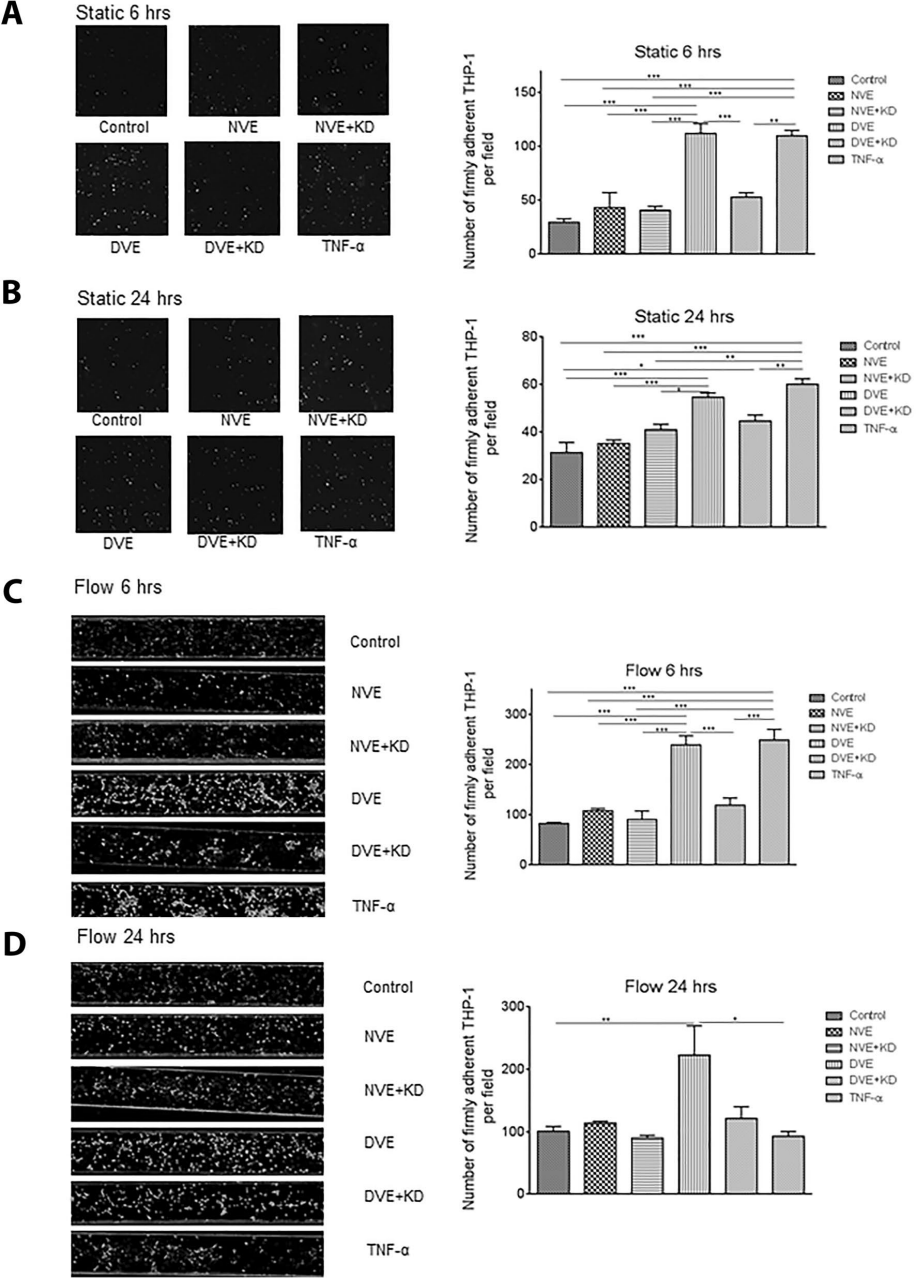
Exposure of HCAECs to DVEs promotes increased adhesion of THP-1 cells under both static and flow conditions. **A**, **B** Number of firmly adherent THP-1 cells on HUVEC under static conditions following 6 (**A**) and 24 (**B**) hours of treatment. **C**, **D** Number of firmly adherent THP-1 cells on HUVEC under shear flow conditions following 6 (**C**) and 24 (**D**) hours of treatment. *p < 0.05, **p < 0.01, ***p < 0.001

Similarly, HCAECs exposed for 6 h to exosomes under static conditions, exhibited a significant increase in the number of adherent THP-1 cells in the DVE and TNF-α groups (112 ± 9, 110 ± 5) as compared to control (30 ± 3), NVE (43 ± 13), NVE + KD (41 ± 4), and DVE + KD (53 ± 4) (Fig. 3A). Exposure for 24 h produced a similar but attenuated effect (Fig. 3B) with the DVE group exhibiting significantly greater THP-1 cell adhesion (55 ± 2) compared to control (31 ± 4), NVE (35 ± 1), and NVE + KD (41 ± 2). Under flow conditions, THP-1 cell attachment to HCAECs was significantly higher in the DVE (239 ± 18) and TNF-α (248 ± 21) groups compared to control (83 ± 2), NVE (108 ± 5), NVE + KD (91 ± 17), and DVE + KD (119 ± 14) (Fig. 3C). This effect was sustained at 24 h with THP-1 cell attachment in the DVE group significantly elevated compared to control (222 ± 47 vs. 101 ± 8) (Fig. 3D). In contrast to HUVECs, the effect of TNF- α was not prolonged out to 24 h. These data suggest that the effect of DVEs on HCAECs is more prolonged especially under flow conditions.

### DVEs increase adhesion molecule expression on endothelial cells

**Fig. 4:**
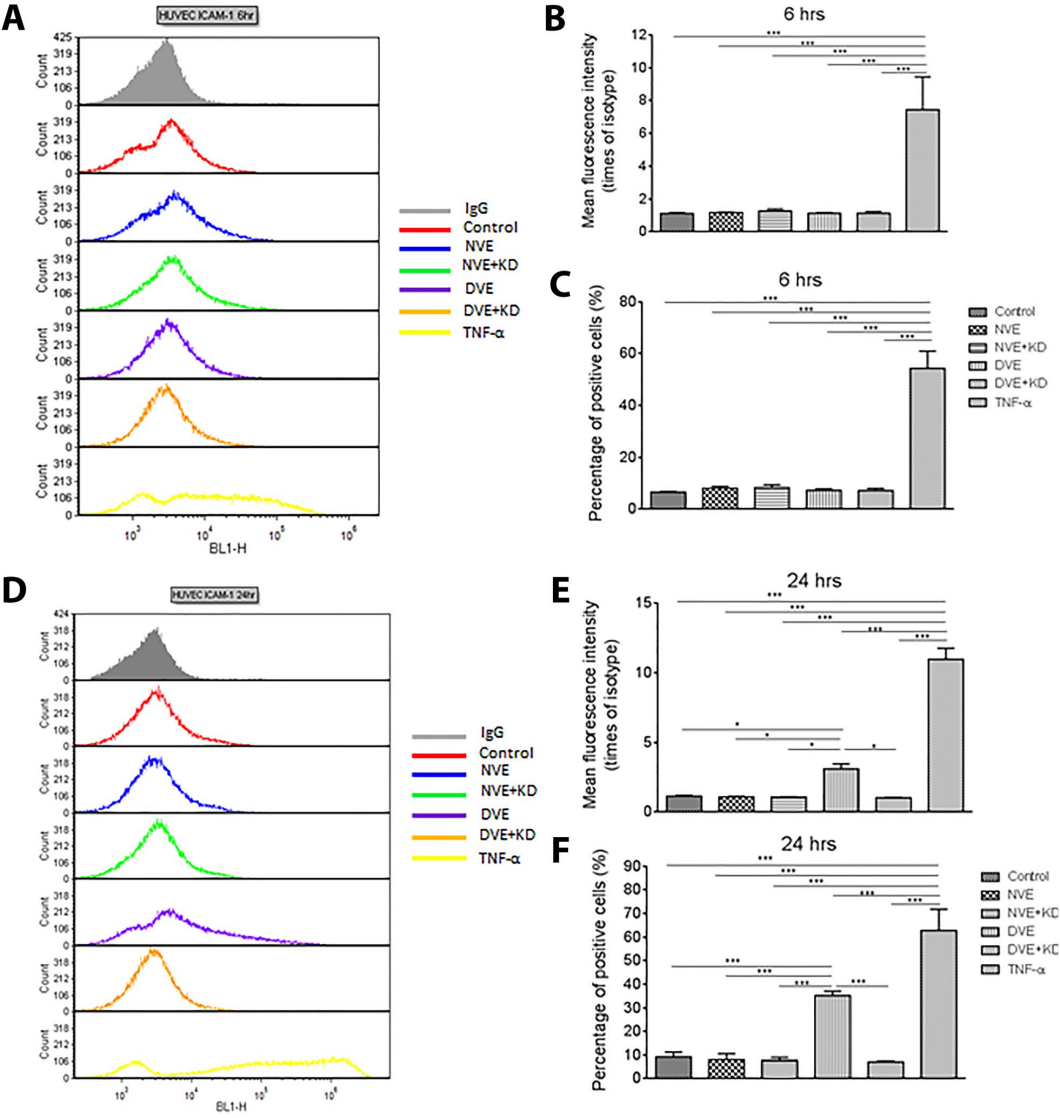
Exposure of HUVECs to DVEs promotes increased surface expression of ICAM-1. **A** Histograms of cell surface expression of ICAM-1 expression following 6 h of treatment. **B** Fluorescence intensity of ICAM-1 antibody relative to the isotype control (mean ± SEM, n = 3). **C** Percentage of HUVECs with positive expression of ICAM-1 (mean ± SEM, n = 3). **D** Histograms of cell surface expression of ICAM-1 expression following 24 h of treatment. **E** Fluorescence intensity of ICAM-1 antibody relative to the isotype control following 24 h of treatment (mean ± SEM, n = 3). **F** Percentage of HUVECs with positive expression of ICAM-1 following 24 h of treatment (mean ± SEM, n = 3). *p < 0.05, **p < 0.01, ***p < 0.001

**Fig. 5:**
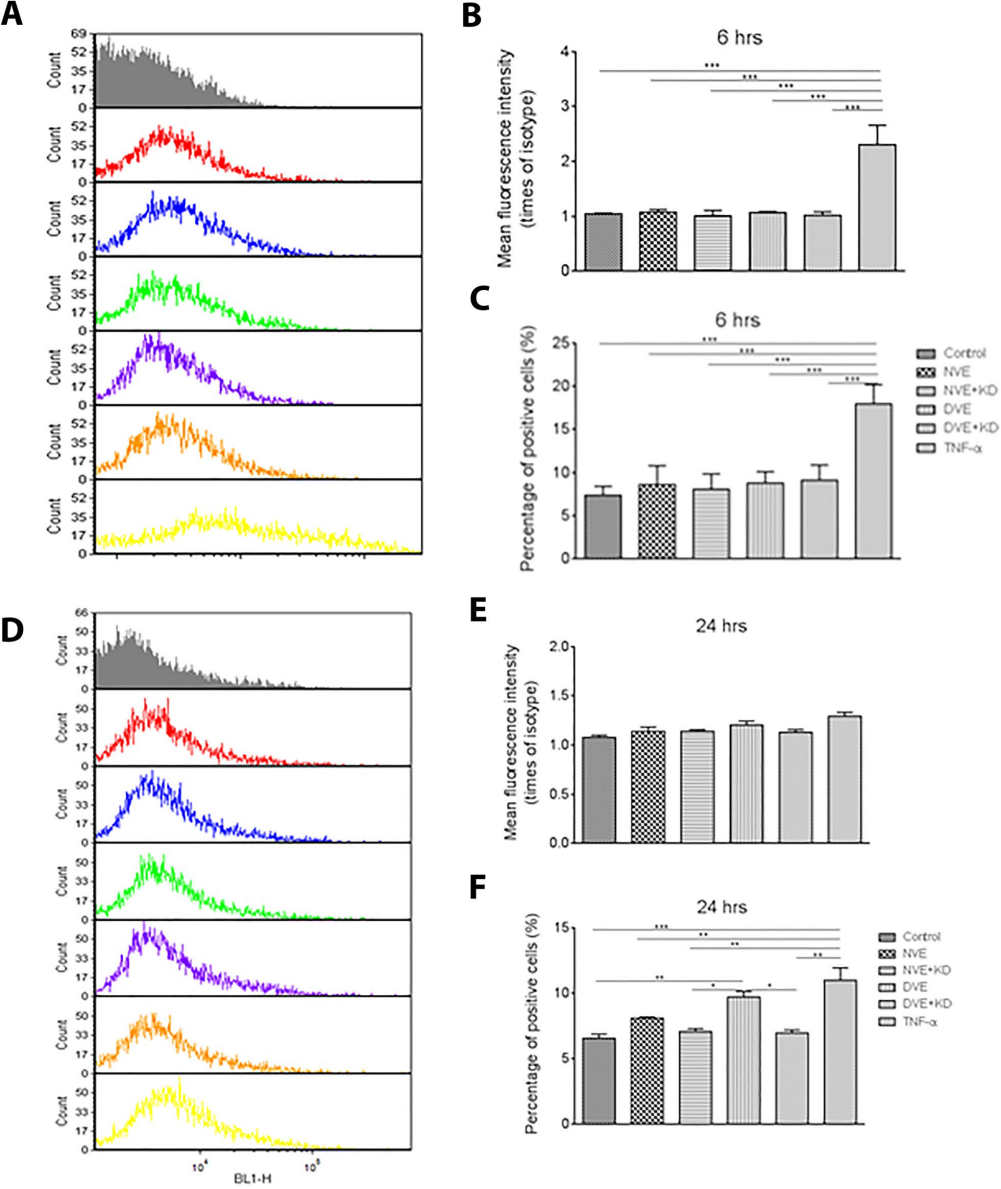
Exposure of HCAECs to DVEs promotes increased surface expression of ICAM-1. **A** Histograms of cell surface expression of ICAM-1 expression following 6 h of treatment. **B** Fluorescence intensity of ICAM-1 antibody relative to the isotype control (mean ± SEM, n = 3). **C** Percentage of HCAECs with positive expression of ICAM-1 (mean ± SEM, n = 3). **D** Histograms of cell surface expression of ICAM-1 expression following 24 h of treatment. **E** Fluorescence intensity of ICAM-1 antibody relative to the isotype control following 24 h of treatment (mean ± SEM, n = 3). **F** Percentage of HCAECs with positive expression of ICAM-1 following 24 h of treatment (mean ± SEM, n = 3). *p < 0.05, **p < 0.01, ***p < 0.001

We next assessed EC activation by NVEs and DVEs by measuring the cell surface expression of adhesion molecules following incubation with DVEs and NVEs. In HUVECs, no change in cell surface expression of ICAM-1, measured as mean fluorescence intensity, and the number of ICAM-1 positive cells was seen following 6 h of exposure to DVEs and NVEs (Fig. 4A–C). Both cell surface ICAM-1 expression and the number of cells positive for ICAM-1 was significantly elevated following 24 h of exposure to DVEs (Fig. 4D–F). In contrast, 6 h incubation with DVEs significantly increased the percentage of HUVECs positive for VCAM-1 (Additional file 1: Fig. S1A-C) as compared to control (4.0% ± 0.4%) and NVEs (3.3% ± 0.6%). This effect is lost at 24 h (Additional file 1: Fig. S1D–F). HCAECs exhibited a similar increase in the percentage of positive ICAM-1 positive cells following exposure to DVEs for 24 h (Fig. 5) but did not exhibit an increase in VCAM-1 staining (Additional file 1: Fig. S2). Importantly, knockdown of miR-221/222 in the source VSMCs abolished the increase in ICAM-1 and VCAM-1 expression induced by DVE exposure in both HUVECs and HCAECs. These data suggest transfer of miR-221/222 by DVEs promotes increased monocyte adhesion to ECs through increases in cell surface ICAM-1.

**Fig. 6:**
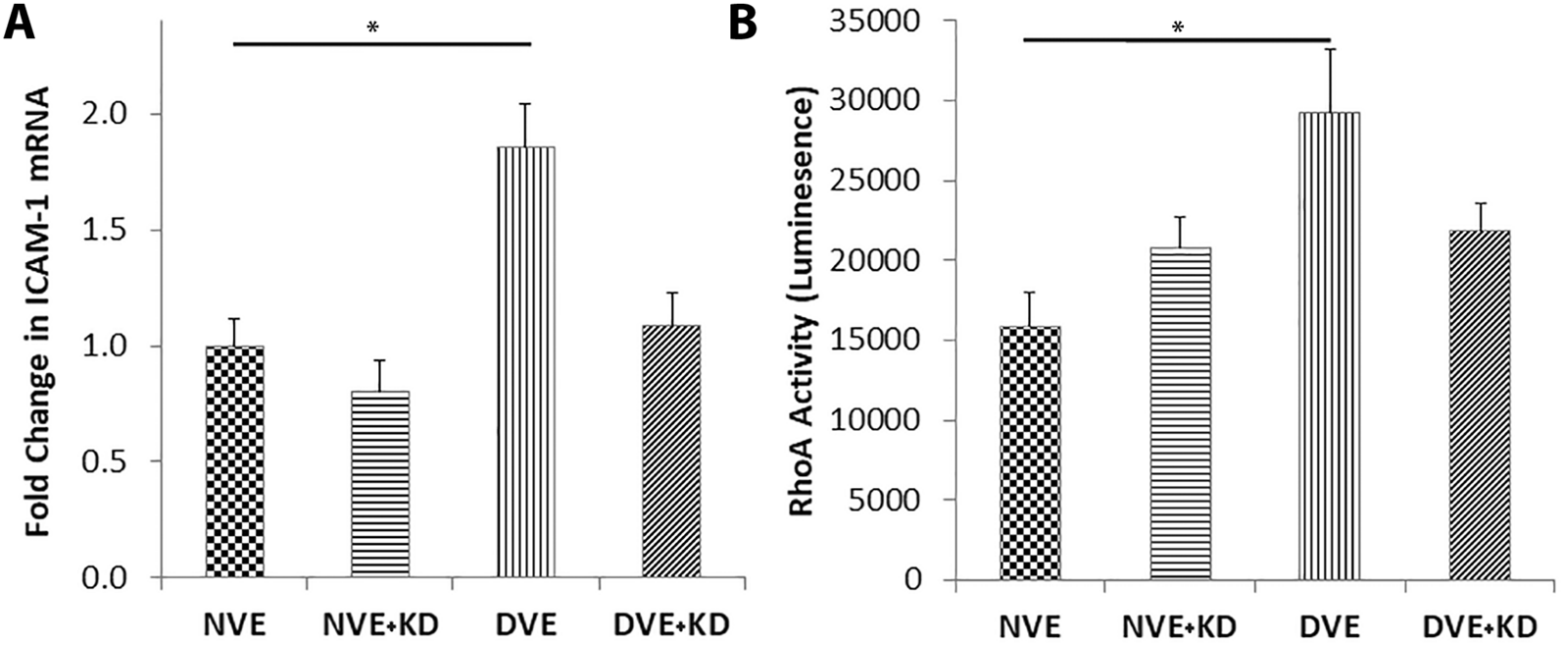
Exposure of HCAECs to DVEs promotes increased surface expression of ICAM-1 and RhoA activity in a miR-221/222 dependent manner. **A** Expression of mRNA encoding ICAM-1 in HCAECS treated with exosomes from VSMCs of non-diabetic (NVE) and diabetic (DVE) origin treated with non-targeting siRNA or miR-221/222 targeting siRNA (+KD). **B** RhoA activity in HCAECS treated with exosomes from VSMCs of non-diabetic (NVE) and diabetic (DVE) origin treated with non-targeting siRNA or miR-221/222 targeting siRNA (+KD). Data is expressed as the mean ± SEM. *p < 0.05

As HCAECs are derived from a vessel known to develop atherosclerosis while HUVECs are not, we now focused on regulation of ICAM-1 in HCAECs. To determine whether the increase in ICAM-1 on the cell surface is due increased expression, we measured mRNA encoding ICAM-1 in HCAECs incubated with DVEs and NVEs. DVE exposure produced a significant increase in ICAM-1 mRNA compared to NVE exposure (Fig. 6A), suggesting increased expression rather a transfer of ICAM-1 protein. miR-221/222 target p27^Kip1^ in ECs and we have previously reported that p27^Kip1^ inhibits RhoA activity in ECs [14, 18]. Loss of inhibition of RhoA by p27^Kip1^ results in increased adhesion molecule expression and atherosclerotic lesion development in the ApoE -/- mouse on a Western diet [23]. HCAECs exposed to DVEs for 18 h exhibited a significant increase in RhoA activity compared to HCAECs exposed to NVEs, NVE + KDs, and DVE + KDs (Fig. 6B). Thus, increased RhoA activity due to miR-221/222 targeting of p27^Kip1^ may represent a potential mechanism for elevated expression of adhesion molecules in ECs.

### DVEs promote M1 polarization of macrophages

**Fig. 7:**
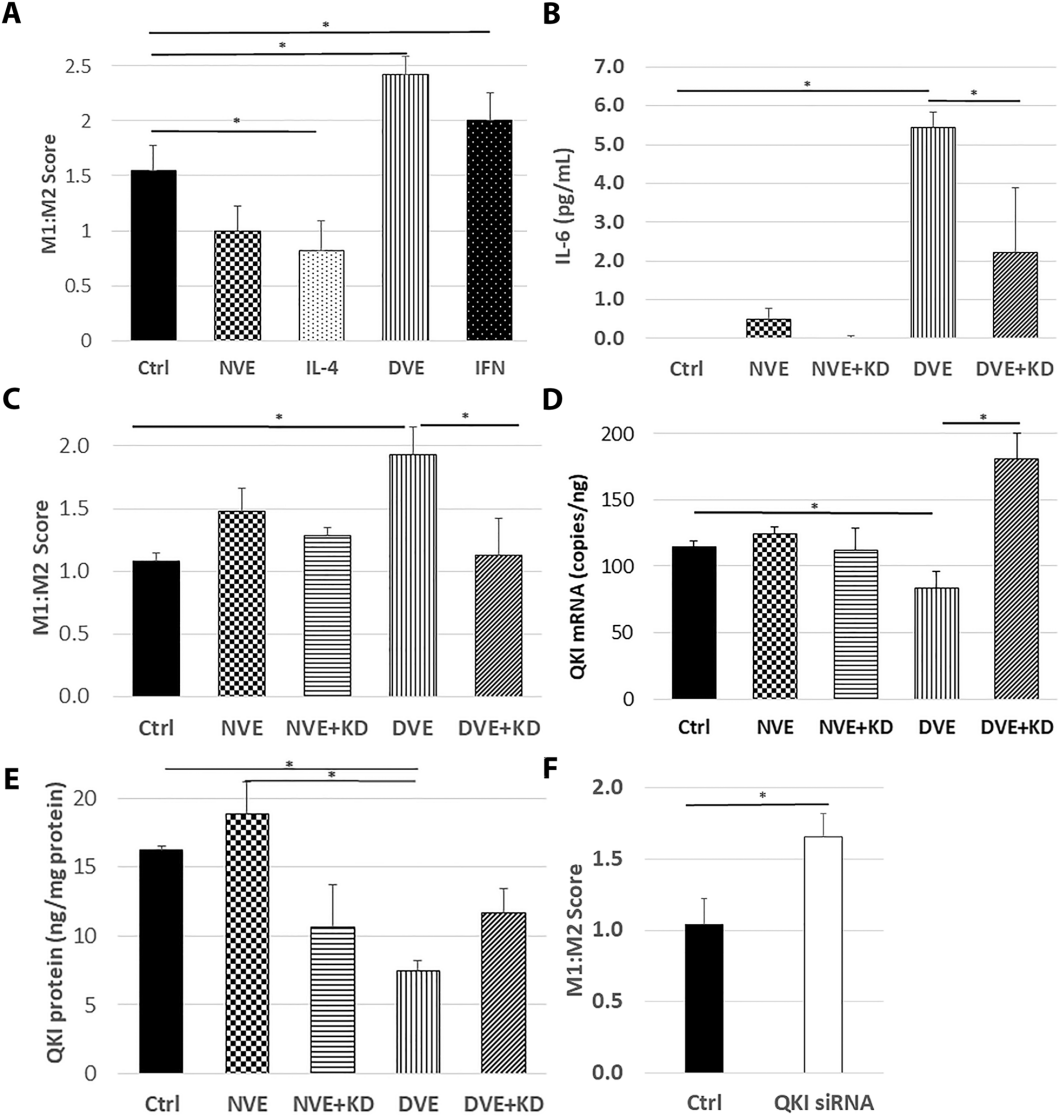
Exposure of CD14 + monocytes to DVEs promotes M1 macrophage polarization in a miR-221/222 dependent manner. **A** M1:M2 score for macrophages treated with vehicle (Ctrl), NVEs, the M2 phenotype inducer, IL-4, DVEs, and the M2 phenotype inducer, interferon-γ. **B** M1:M2 score for macrophages treated with vehicle (Ctrl), NVEs, NVE+KDs, DVEs, and DVE+KDs. **C** IL-6 concentrations in conditioned media of CD14 + macrophages treated with vehicle (Ctrl), NVEs, NVE+KDs, DVEs, and DVE+KDs. **D** *QKI* copies in CD14 + macrophages treated with vehicle (Ctrl), NVEs, NVE+Ds, DVEs, and DVE+KDs. **E** *QKI* copies in CD14 + macrophages treated with vehicle (Ctrl), NVEs, NVE+KDs, DVEs, and DVE+KDs. **F** M1:M2 score for macrophages treated with non-targeting (Ctrl) or *QKI* targeting siRNA.*p < 0.05

Macrophages are a key driver of atherosclerotic plaque formation that exist in multiple phenotypes including the M1 (pro-inflammatory) and M2 (anti-inflammatory) phenotypes. M1 macrophages are associated more advanced lesions, while M2 is associated with early lesions and plaque regression [24–26]. We measured macrophage polarization using an M1:M2 score based on RNA associated with the individual phenotypes [20, 21]. Macrophages incubated with DVEs exhibited a significantly increased M1:M2 score compared to control (1.5 ± 0.2 vs. 2.4 ± 0.2, p < 0.05, Fig. 7A). Macrophages treated with the M2 phenotypes inducer, interleukin-4 (IL-4), exhibited a significantly lower M1:M2 score (0.8 ± 0.3) and those treated with the M1 inducer, interferon-γ (IFN), exhibited a significantly higher M1:M2 score (2.0 ± 0.3). Furthermore, incubation with DVEs produced a significant increase in the secretion of interleukin-6 (IL-6) by macrophages compared to control (5.4 ± 0.4 pg/mL vs. 0.0 ± 0.1 pg/mL, p <0.001, Fig. 7B).  The increase in M1:M2 score in response to DVEs is miR-221/222 dependent as, in a separate study, exposure to DVEs resulted in a significantly higher M1:M2 score (1.9 ± 0.2) than control (1.1 ± 0.1) and DVE + KDs (1.1 ± 0.3) treated cells (Fig. 7C). This increase in IL-6 secretion was also significantly reduced when macrophages were incubated with DVE + KD (5.4 ± 0.4 pg/mL vs. 2.2 ± 1.7 pg/mL, p < 0.01). Macrophages incubated with NVEs also exhibited a mild increase in IL-6 secretion (0.5 ± 0.5 pg/mL) that was not seen with NVE + KDs and may be due the transfer of the lower level of miR-221/222 present in NVEs.

To identify a mechanism of action for mir-221/222 to promote M1 polarization, we performed an in silico analysis combining predicted targets of each miRNA (miRDB.org) with a publicly available expression data (GSE5099) obtained from M1 and M2 macrophages [27, 28] and identified *QKI* as a candidate target of miR-221/222 that was reduced in M1 macrophages. Following incubation with DVEs, *QKI* was significantly reduced compared to control (83 ± 13 vs. 115 ± 4 copies/ng, p < 0.05, Fig. 7D). QKI protein was also significantly reduced compared to control following incubation with DVEs (7.5 ± 0.7 vs. 16.2 ± 0.3 ng/mg, p < 0.05, Fig. 7E). Knockdown of miR-221/222 in the DVEs restored both *QKI* mRNA and protein expression (180 ± 18 copies/ng and 11.6 ± 18 ng/mg, respectively). Additionally, knockdown of *QKI* in macrophages with siRNA leads to a significant increase in the M1:M2 score (1.0 ± 0.2 vs. 1.7 ± 0.2, p < 0.05, Fig. 7F). This suggests downregulation of *QKI* mRNA by miR-221/222 as a mechanism underlying the ability of DVEs to promote M1 polarization of macrophages.

### DVEs promote atherosclerotic plaque formation

**Fig. 8:**
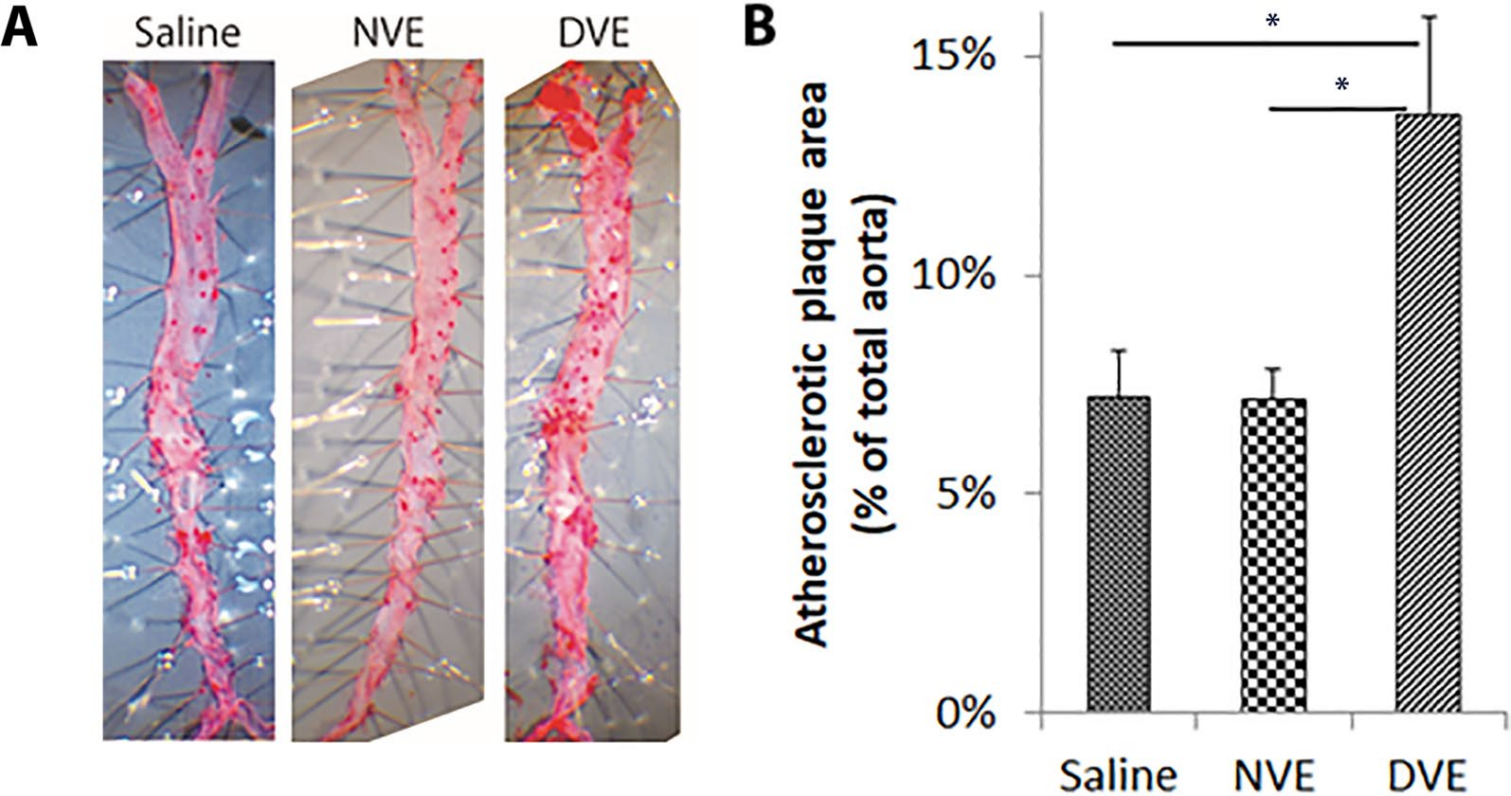
DVEs, but not NVEs, promote atherosclerotic lesion formation. **A** Representative images of Oil Red O staining from ApoE^−/−^ mice maintained on a Western diet (42% fat) and receiving an injection of 5 × 10.^7^ DVEs (n = 5) or NVEs (n = 5) or saline (n = 5) every other day for 6 weeks. **B** Mean atherosclerotic plaque area in ApoE^−/−^ mice maintained on a Western diet (42% fat) and receiving an injection of 5 × 107 DVEs (n = 5) or NVEs (n = 5) or saline (n = 5) every other day for 6 weeks. *p < 0.05

Having shown that exosomes can facilitate transfer of miRNAs that promote vascular inflammation in vitro from VSMCs to adjacent cells, we next asked if these exosomes can impact atherosclerotic lesion formation. We confirmed that intravenous administration of VEs results in transfer of miRNAs to the vasculature by detecting a *C. elegans* specific miRNA, cel-miR-39, in the vasculature after tail vein injection of VEs isolated from VSMCs that were transfected with miR-39 (data not shown). Next, ApoE^−/−^ mice on a western diet were divided into three 6 week intravenous treatment groups: (1) a control group receiving saline, (2) a group receiving NVEs (5 × 10^7^ exosomes/dose, q.o.d.), and (3) a group receiving DVEs (5 × 10^7^ exosomes/dose, q.o.d.). Treatment with DVEs, but not Saline or NVEs, for resulted in a doubling of the atherosclerotic plaque area in the aorta (Fig. 8). Thus, administration of DVEs, but not NVEs, results in increased atherosclerotic plaque formation.

## Discussion

We previously demonstrated that miR-221/222 are elevated in the arteries of diabetic animals and patients, both in the presence and absence of atherosclerotic disease [15–17]. In VSMCs, this increase in miR-221/222 accelerates intimal thickening through reductions in the cell cycle checkpoint protein, p27^Kip1^ [17]. Our results demonstrate that the increase in miR-221/222 induced in VSMCs by diabetes is also seen in DVEs. Exosomes isolated from VSMCs, macrophages and ECs have been demonstrated to have pro- and anti-atherosclerotic effects based on the phenotype of the source cells [5, 14, 29–32]. Here we present a novel paracrine signaling mechanism underlying the cardiovascular complications of diabetes, where DVEs promote EC activation and M1 monocyte polarization.

Our results indicate that the DVE exposure promotes endothelium activation, induces monocyte adhesion and increases the expression of EC adhesion molecules. The monocyte adhesion to HUVECs or HCAECs activated by DVE was elevated at 6 h, but at 24 h, it was reduced in HCAECs and eliminated for HUVECs. Neither EC type exhibited changes in ICAM-1 expression at 6 h, but both exhibited significant expression at 24 h. VCAM-1 expression was elevated in HUVECs but not HCAECs at 6 h, while at 24 h only HCAECs demonstrated an increase. Based on the short time frame, these results point to HUVECs and HCAECs being activated by yet unidentified proteins in DVEs promoting acute increases in VCAM-1 expression in HUVECs and strong monocyte adhesion to both HUVECs and HCAECs. The miR221/222 dependent effect was pronounced at 24 h and was stronger in HCAECs than in HUVECs. In particular, it caused the increased expression of both ICAM-1 and VCAM-1 in HCAECs and increased expression of ICAM-1 in HUVECs. The monocyte adhesion to HCAECs was stronger under flow than static conditions at 24 h of DVE exposure because of the increased expression of both VCAM-1 and ICAM-1. Flowing monocytes need to be captured by and rollon ECs before they were anchored to ECs by ICAM-1 binding to lymphocyte function-associated antigen-1 (LFA-1/αLβ2 integrin) and macrophage-1 antigen (Mac-1/αMβ2 integrin) [33]. Binding VCAM-1 to very late antigen-4 (VLA-4/α4β1 integrin) mediates monocyte rolling [34]. The similar difference between flow and static adhesion to HUVECs at 6 h can be explained by the same effect.

Our study demonstrates an increase in the mRNA encoding ICAM-1. In contrast, Rautou et al*.* demonstrated that microparticles isolated from carotid artery plaques promote monocyte adhesion to HUVECs through an immediate increase in cell surface ICAM-1 via transfer of ICAM-1 protein without changes in ICAM-1 expression [35]. The microparticles in that study would have included not only exosomes, but also apoptotic bodies and other microparticles. It would have also included microparticles from the multiple cell sources in the complex environment of an atherosclerotic plaque. Thus, the transfer of ICAM-1 protein may occur from other cells than VSMCs or particles other than exosomes, and likely represents a different mechanism than presented here.

Our finding of an increase in ICAM-1 mRNA in unstimulated ECs following DVE exposure are in contrast to previous reports showing miR-221/222 target ICAM-1 mRNA (*ICAM1)* and reduce its expression [36, 37]. The function of miR-221/222, as all miRNAs, is dictated by the relative concentration of their targets [14]. The studies reporting that miR-221/222 target *ICAM1* were conducted in HUVECs stimulated with either fetal bovine serum containing growth media or TNF-α, both of which will may reduce other targets of miR-221/222, especially those associated with growth arrest such as *CDKN1B*, which encodes p27.^Kip1^. The predicted affinity of miR-221/222 to *ICAM1* as calculated by TargetScan (https://www.targetscan.org) is significantly less than that of *CDKN1B* [38]. The studies presented here were conducted in growth arrested confluent ECs, where the expression of *CDKN1B* is elevated. Thus, the difference in the effect of miR-221/222 on ICAM-1 expression may reflect differences in the expression of competing targets due to differing states of the ECs. The effect of TNF-α on miR-221/222 is also controversial as it has been found to both suppress and enhance miR-221/222 [37, 39].

Activation of RhoA promotes increased ICAM-1 expression and monocyte adhesion in vascular ECs [40, 41]. p27^Kip1^ directly binds to RhoA in the cytoplasm preventing its activation by guaninine-nucleotide exchange factors [42]. We and others have previously shown that loss of p27^Kip1^ in HUVECs and HCAECs leads to increased RhoA activity [18, 43]. Thus, repression of p27^Kip1^ expression through increased miR-221/222 can drive increased ICAM-1 expression through increased activation of RhoA. We show that exposure to DVEs is associated with an increase in RhoA activity in ECs in a mir-221/222 dependent manner, suggesting enhanced RhoA activity as the link between DVEs and EC activation.

Monocytes that adhere to the endothelium diapedes into the vessel wall and become macrophages. Macrophages within the vessel wall exist in a variety of phenotypes in general ranging from an inflammatory state with a high phagocytic activity (M1) to a resolving phenotype (M2). Atherosclerotic plaque development is associated with a predominance of resident M1 macrophages [24]. Our in silico analysis of transcripts that were differentially expressed between M1 and M2 macrophages identified *QKI* as a potential target of miR-221/222 regulating macrophage phenotype. *QKI* has been demonstrated to be a direct target of miR-221/222 [44]. Silencing of *QKI* in macrophages is associated with an increase in the M1 phenotype [45]. Here we demonstrate that macrophages exposed to DVEs exhibit a down-regulation of QKI mRNA and protein coupled with polarization toward an M1 phenotype.

Together, our in vitro studies demonstrate that DVEs promote mechanisms that will produce an increase in inflammation in an artery. Our in vivo study confirmed that injection of DVEs into a non-diabetic mouse model drives an increase in atherosclerotic plaque formation. In a similar fashion, Hergenreider et al*.* demonstrated that laminar flow promoted an increase in miR-143 and -144 in EC-derived exosomes and that these reduced atherosclerotic plaque formation. Zheng et al*.* demonstrated that overexpression of Kruppel-like factor 5 in VSMCs produced VEs that promote atherosclerosis. These studies demonstrate a clear role for exosomes in atherosclerosis. Exosomes isolated from diabetic cardiomyocytes and insulin resistant adipocytes inhibit angiogenesis, demonstrating that the presence of diabetes can alter the content of exosomes [46, 47]. Here we extend these findings to demonstrate a role for exosomes in the development of the cardiovascular complications of diabetes.

In summary, diabetes promotes an increase of miR-221/222 in the exosomes derived from VSMCs and that these exosomes promote atherosclerotic plaque development by increasing endothelial activation and inflammatory polarization of macrophages. We have previously demonstrated the potential utility of diagnostics that exploit an increase serum miR-221 to identify those subjects at greater risk of heart attacks and strokes [12]. These data support the need for studies into therapies that prevent the increase in miR-221/222 in VSMCs of diabetic patients for prevention of the cardiovascular complications of diabetes.

## Supplementary Information

**Additional file 1: Figure S1. **Exposure of HUVECs to DVEs promotes increased surface expression of VCAM-1. A: Histograms of cell surface expression of VCAM-1 expression following 6 hours of treatment. B*: *Fluorescence intensity of VCAM-1 antibody relative to the isotype control. C: Percentage of HUVECs with positive expression of VCAM-1. D: Histograms of cell surface expression of VCAM-1 expression following 24 hours of treatment. E: Fluorescence intensity of VCAM-1 antibody relative to the isotype control following 24 hours of treatment. F: Percentage of HUVECs with positive expression of VCAM-1 following 24 hours of treatment. * p < 0.05, ** p < 0.01, *** p < 0.001. **Figure S2. **Exposure of HCAECs to DVEs promotes increased surface expression of VCAM-1. A: Histograms of cell surface expression of VCAM-1 expression following 6 hours of treatment. B: Fluorescence intensity of VCAM-1 antibody relative to the isotype control. C: Percentage of HCAECs with positive expression of VCAM-1. D: Histograms of cell surface expression of VCAM-1 expression following 24 hours of treatment. E: Fluorescence intensity of VCAM-1 antibody relative to the isotype control following 24 hours of treatment. F: Percentage of HCAECs with positive expression of VCAM-1 following 24 hours of treatment. * p < 0.05, ** p < 0.01, *** p < 0.001.

## Data Availability

All data generated or analyzed during this study are included in this published article.
