# Supplementary material for: Diabetes is accompanied by secretion of pro-atherosclerotic exosomes from vascular smooth muscle cells
Source: Cardiovasc Diabetol. 2023 May 13;22:112. doi: 10.1186/s12933-023-01833-4 (PMC10183121; doi:10.1186/s12933-023-01833-4)
Supplement: Supplementary file 1 — Additional file 1: Figure S1. Exposure of HUVECs to DVEs promotes increased surface expression of VCAM-1. A: Histograms of cell surface expression of VCAM-1 expression following 6 hours of treatment. B: Fluorescence intensity of VCAM-1 antibody relative to the isotype control. C: Percentage of HUVECs with positive expression of VCAM-1. D: Histograms of cell surface expression of VCAM-1 expression following 24 hours of treatment. E: Fluorescence intensity of VCAM-1 antibody relative to the isotype control following 24 hours of treatment. F: Percentage of HUVECs with positive expression of VCAM-1 following 24 hours of treatment. * p < 0.05, ** p < 0.01, *** p < 0.001. Figure S2. Exposure of HCAECs to DVEs promotes increased surface expression of VCAM-1. A: Histograms of cell surface expression of VCAM-1 expression following 6 hours of treatment. B: Fluorescence intensity of VCAM-1 antibody relative to the isotype control. C: Percentage of HCAECs with positive expression of VCAM-1. D: Histograms of cell surface expression of VCAM-1 expression following 24 hours of treatment. E: Fluorescence intensity of VCAM-1 antibody relative to the isotype control following 24 hours of treatment. F: Percentage of HCAECs with positive expression of VCAM-1 following 24 hours of treatment. * p < 0.05, ** p < 0.01, *** p < 0.001. [file 12933_2023_1833_MOESM1_ESM.pdf]

Additional file 1: Material

for

Diabetes is accompanied by secretion of pro-atherosclerotic exosomes from vascular smooth muscle cells by

Heng Yu[a], Hunter F. Douglas[b], Donald Wathieu[b], Ryan A. Braun[b], Christine Edomwande[b], Daniel J. Lightell, Jr.[b,], Thaidan Pham[b], Natasha C. Klingenberg[b], Shelia Pugh Bishop[b], Damir B. Khismatullin[a], and T. Cooper Woods[b*]

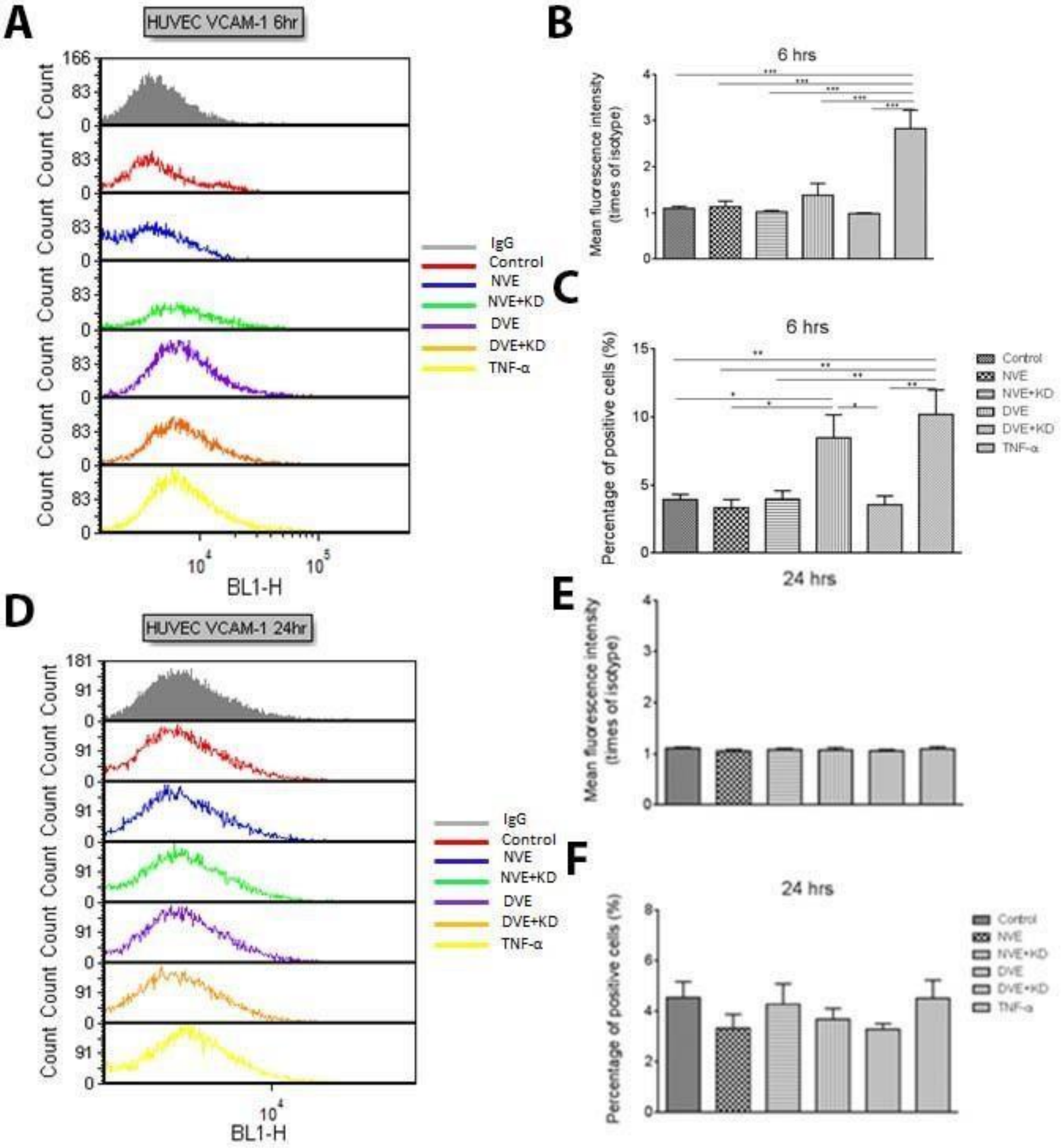

Figure S1 – Exposure of HUVECs to DVEs promotes increased surface expression of VCAM-1. *A*: Histograms of cell surface expression of VCAM-1 expression following 6 hours of treatment. *B:* Fluorescence intensity of VCAM-1 antibody relative to the isotype control (mean ± SEM, n = 3). *C:* Percentage of HUVECs with positive expression of VCAM-1 (mean ± SEM, n = 3). *D*: Histograms of cell surface expression of VCAM-1 expression following 24 hours of treatment. *E:* Fluorescence intensity of VCAM-1 antibody relative to the isotype control following 24 hours of treatment (mean ± SEM, n = 3). *F:* Percentage of HUVECs with positive expression of VCAM-1 following 24 hours of treatment (mean ± SEM, n = 3). * $p < 0.05$, ** $p < 0.01$, *** $p < 0.001$.

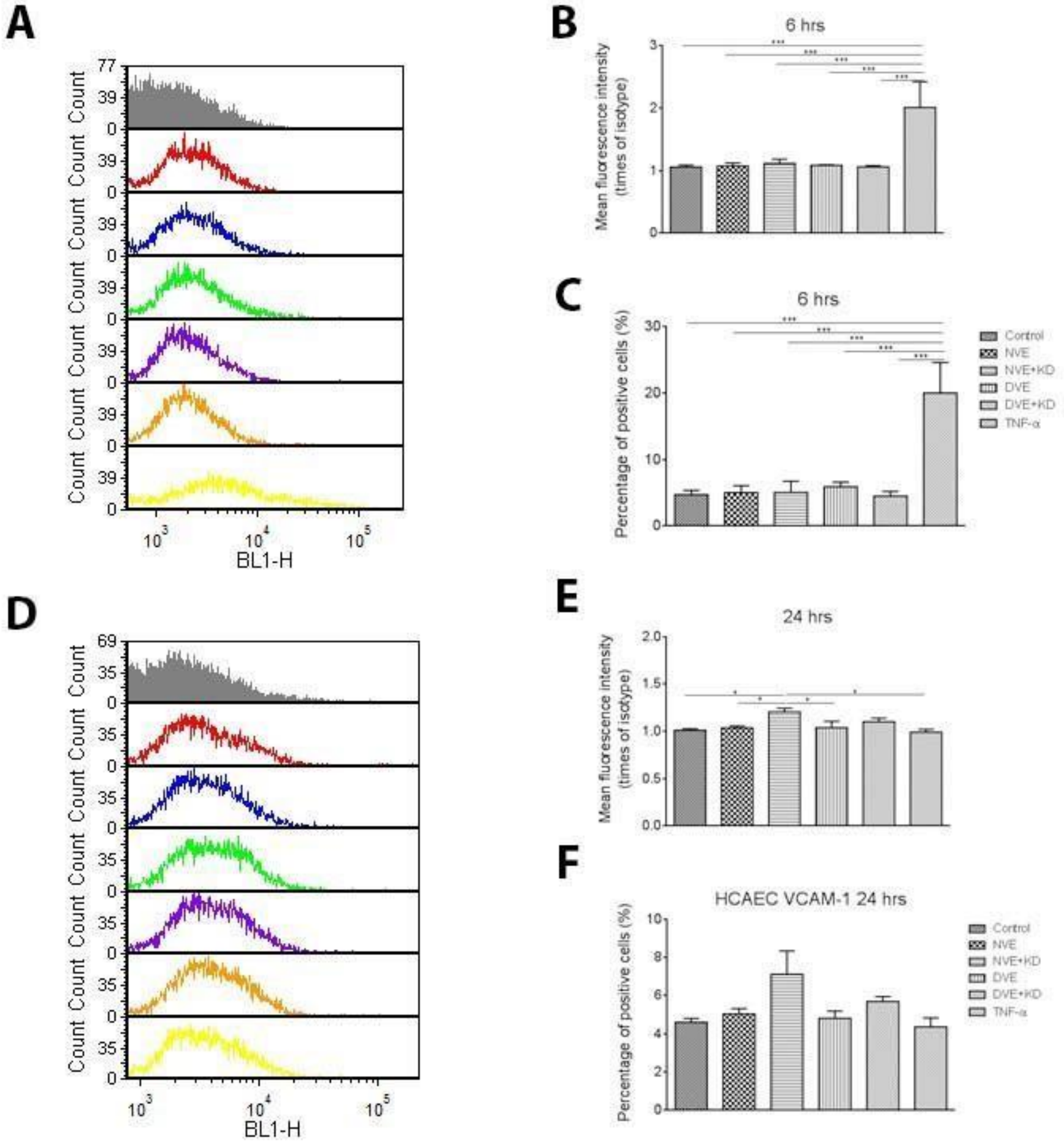

Figure S2 – Exposure of HCAECs to DVEs promotes increased surface expression of VCAM-1. *A*: Histograms of cell surface expression of VCAM-1 expression following 6 hours of treatment. *B:* Fluorescence intensity of VCAM-1 antibody relative to the isotype control (mean ± SEM, n = 3). *C:* Percentage of HCAECs with positive expression of VCAM-1 (mean ± SEM, n = 3). *D*: Histograms of cell surface expression of VCAM-1 expression following 24 hours of treatment. *E:* Fluorescence intensity of VCAM-1 antibody relative to the isotype control following 24 hours of treatment (mean ± SEM, n = 3). *F:* Percentage of HCAECs with positive expression of VCAM-1 following 24 hours of treatment (mean ± SEM, n = 3). * $p < 0.05$, ** $p < 0.01$, *** $p < 0.001$.
